# Supplementary material for: Fortification of Bread with Carob Extract: A Comprehensive Study on Dough Behavior and Product Quality
Source: Foods. 2025 May 20;14(10):1821. doi: 10.3390/foods14101821 (PMC12111294; doi:10.3390/foods14101821)
Supplement: Supplementary file 1 [file foods-14-01821-s001.zip › foods-3634668-supplementary.pdf]

Supplementary material

1)

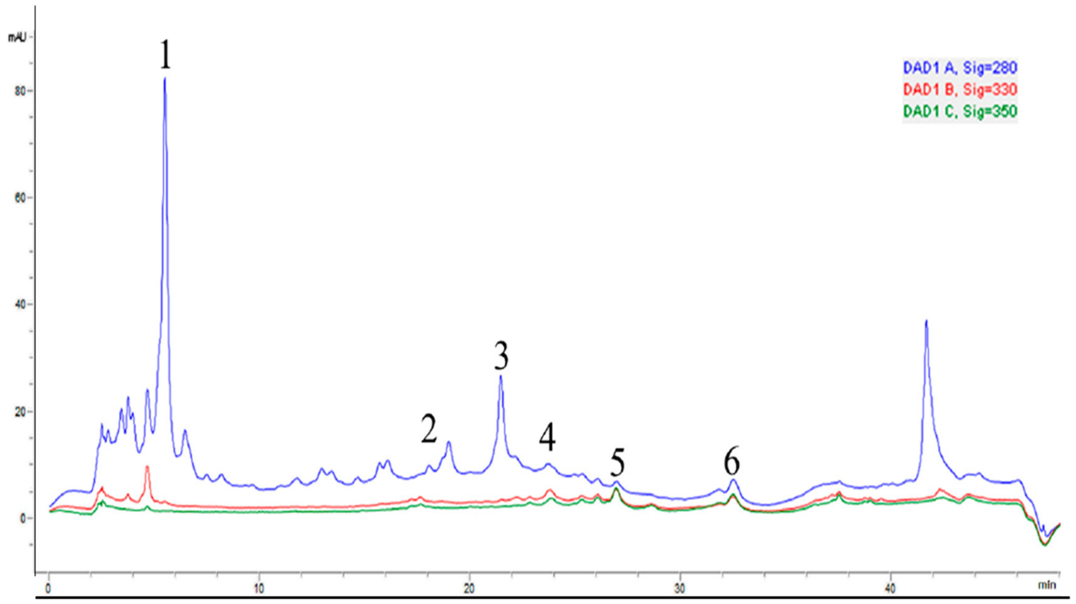

2)

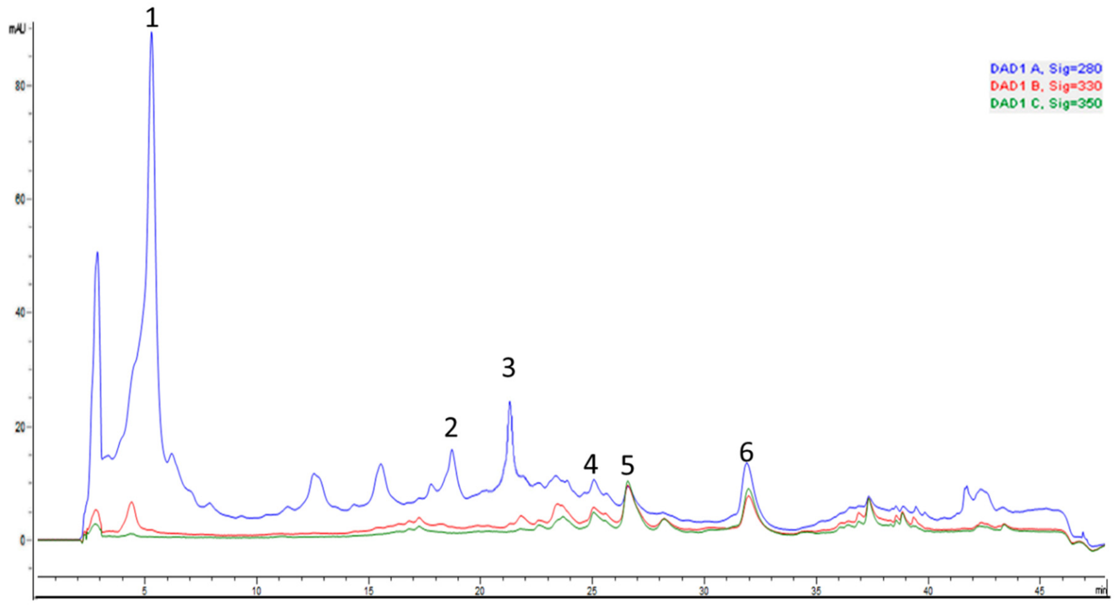

Figure S1. Chromatogram of 1) carob liquid extract and 2) powdered carob extract with detection at 280 nm, 330 nm and 350 nm: 1-Gallic acid, 2-Quercetin, 3-Caffeic acid, 4-Chlorogenic acid, 5-Rutin, 6-Quercitrin.

Table S1. Limit of detection (LOD) and limit of quantification (LOQ) for investigated compounds.

Analyte	LOD (µg/g of dry extract)	LOQ (µg/g of dry extract)
trans-cinnamic acid	1.5	5.0
caffeic acid	1.5	10.0
p-coumaric acid	1.5	10.0
Quercetin	1.5	10.0
chlorogenic acid	6.5	15.0
rosmarinic acid	8.0	25.0
ferulic acid	4.5	12.0
gallic acid	2.5	15.0
Rutin	8.0	25.0
Quercitrin	6.5	25.0

Table S2. Sensory evaluation results by individual evaluators.

Sample*	External appearence	Crumb structure	Smell	Taste
Evaluator 1				
control	20	24	14	21
PCE1	20	24	16	27
PCE3	20	27	20	30
PCE5	20	24	20	30
Evaluator 2				
control	20	24	12	18
PCE1	20	24	18	27
PCE3	20	27	20	30
PCE5	20	27	20	30
Evaluator 3				
control	20	27	12	18
PCE1	20	24	20	24
PCE3	20	27	20	30
PCE5	20	27	20	30
Evaluator 4				
control	20	24	14	21
PCE1	20	24	18	27
PCE3	20	27	20	30
PCE5	20	30	20	30
Evaluator 5				
control	20	21	14	27
PCE1	20	24	18	30
PCE3	20	24	20	30
PCE5	20	27	20	30
Evaluator 6				
control	20	24	18	21
PCE1	20	24	18	27

PCE3	20	30	20	30
PCE5	20	27	20	30

*PCE – powdered carob extract.
